# Evaluation of Utility of the Cement Solidification Process of Waste Ion Exchange Resin

**DOI:** 10.3390/toxics10030120

**Published:** 2022-03-02

**Authors:** Jong-Sik Shon, Hyun-Kyu Lee, Tack-Jin Kim, Jong-Won Choi, Woo-Yeol Yoon, Sang-Bok Ahn

**Affiliations:** 1 Radwaste Management Center, Korea Atomic Energy Research Institute, 111, Daedeok-daero 989beon-gil, Yuseong-gu, Daejeon 34057, Korea; njsshon@kaeri.re.kr (J.-S.S.); ktj@kaeri.re.kr (T.-J.K.); njwchoi@kaeri.re.kr (J.-W.C.); 2 Korea Radioactive Waste Agency, 19, Chunghyocheon-gil, Gyeongju-si 38062, Korea; wyyoon@korad.or.kr (W.-Y.Y.); sbahn@korad.or.kr (S.-B.A.)

**Keywords:** cement solidification, operating conditions, PCP, WAC

## Abstract

The present study aimed to evaluate the utility of the cement solidification process for stably disposing of waste ion exchange resin generated during the treatment of radioactive wastewater. The cement solidification process using the in-drum mixing system was selected to be used for the solidification process of waste ion exchange resins. The disposal safety of waste forms was evaluated according to the waste acceptance criteria (WAC) applicable to domestic waste disposal sites, and the tests were conducted for six test items provided in the WAC. A total of 15 representative samples were collected from the waste-form drums produced using the optimum operating conditions, and their structural stability for disposal considerations was evaluated. In addition, the leaching index of the samples was 11.05, 10.12, 8.39 for Co, Sr, and Cs, respectively, and it was found to exceed 6, the leaching index standard of WAC. The results confirmed that cement waste forms including waste ion exchange resins produced through this process were considered to be conforming to the requirements for disposal safety.

## 1. Introduction

Ion exchange resins are categorized according to the moisture content into as-received resins, damp resins, dewatered resins, and slurry resins. The water uptake of as-received resins is at the level of saturation or lower. Damp resins are those of which only the inside part is saturated with moisture. Damp resins are prepared by removing free-standing water by decompression. Thus, wet resins do not contain free-standing water. The *w*/*c* ratio was determined assuming that the waste resins used in the present study were wet resins. Table 1 presents the classification of ion exchange resins according to the moisture content.

The process control program (PCP) of the solidification process is defined as a set of procedures used to ensure that waste forms that meet all applicable regulatory requirements, including the requirements of disposal sites, can be produced in a consistent manner [1]. Simply put, the program refers to a series of systematic procedures required to produce quality waste forms that can meet all requirements under the country’s laws and regulations on nuclear power, regulatory requirements, and the waste acceptance criteria (WAC) of disposal sites, which is operated by the Korean Radioactive Waste Agency (KORAD).

When selecting a solidification process, the following five factors are generally considered: simplicity, reliability, easy maintenance, minimum radiation exposure, and reasonable installation costs [2,3]. The PCP also involves systematic production procedures because the quality of the resultant waste forms must be maintained in a consistent manner. Requirements to consider when selecting a solidification process are summarized in Table 2.

The cement solidification process using the in-drum mixing system was selected to be used for the solidification process of waste ion exchange resins. The in-drum mixing-based solidification process produces waste forms with high homogeneity, ensures easy quality control, and involves simple processing equipment compared to other solidification processes [3,4]. Notably, this process is suitable for the treatment of a small amount of waste.

Factors to consider in the operation of the cement solidification process include the safety and cost-effectiveness of the resultant waste forms, as well as the characteristics of the selected processes. First of all, waste forms are produced to be safely disposed of at disposal sites. Thus, the safety of produced waste forms must fulfill the WAC of disposal sites, and at the same time, the content of waste in waste forms needs to be increased for improved cost-effectiveness. Different types of waste, such as concentrated liquid waste, waste ion exchange resin, and sludge, are treated in the cement solidification process. Among them, waste ion exchange resins tend to swell and thus can be easily collapsed during water immersion tests [5,6,7]. For that reason, the conduct of water immersion tests is among the most critical factors in producing cement waste forms using waste ion exchange resins. Factors to consider when operating a solidification process facility are summarized in Table 3.

All processes of nuclear facilities are conducted in accordance with the established operative procedures to ensure consistent performance and quality. The utility of the cement solidification process is regularly evaluated with respect to whether its technical specifications are maintained at the levels established when the process was first licensed, as well as whether the quality of its products is maintained.

**Figure 1:**
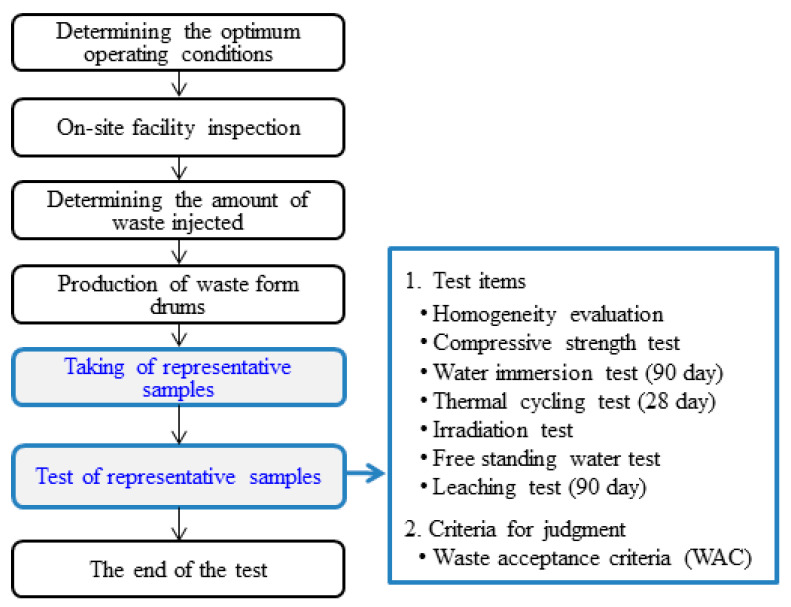
Flowchart and test items for waste forms to assess the utility of the cement solidification process.

These factors are provided in notifications on nuclear power, quality assurance manuals for nuclear facilities, acceptance criteria for low- and intermediate-level radioactive waste, etc. The acceptance criteria for low- and intermediate-level radioactive waste specify the following requirements: (1) If the utility of the process is regularly evaluated every four years; (2) If the properties of waste can be changed by modifying the waste treatment process and procedures; (3) If compressive strength tests are conducted after every ten batches; (4) If all test samples were collected from actual waste drums or fabricated during the actual treatment process [8]. Figure 1 is a flowchart showing the procedures for assessing the utility of the cement solidification process, along with the test items for waste forms.

Representative samples are collected from cured waste-form drums through mechanical coring. Here, mechanical coring is a process in which waste form samples are subjected to various conditions, such as mechanical force, frictional heat, and coolant supply. However, this method often ends up with damaged samples, and such samples cannot properly represent the intrinsic properties of a given waste form [9]. This also means that mechanical coring may affect such properties as free-standing water, compressive strength, leaching behavior of samples. In this regard, mechanical coring of solidified drums is at best the second-best option; it is applied only when waste forms have already been produced, or there is no other method available to collect samples. Accordingly, representative samples need to be collected before cement-waste mixtures harden to properly represent the properties of the corresponding waste-form drum. In the present study, representative samples were collected from waste-cement mixtures within the set time frame, i.e., 3.5 h of the initial setting time, using a specially designed sample collector.

The present study aimed to evaluate the utility of an in-drum mixing-based cementation facility, especially with respect to its ability to produce waste forms that meet the technical specifications previously established when the facility was licensed, and further determine whether the quality of its waste forms fulfills the waste acceptance criteria of disposal sites.

## 2. Materials and Methods

### 2.1. Materials

#### 2.1.1. Cement

In Korea, Portland Cement Type I is typically sold under the name of “Type I Normal Portland Cement”. In the present study, Portland Cement Type I, a commercial product produced by Ssangyong Cement Industrial Co. (Ltd.) (Yeongweol, South Korea), was used in the tests. Cement is categorized into Type I–V according to the weight content of 3CaO·SiO_2_, 2CaO·SiO_2_, 3CaO·Al_2_O_3_, and 4CaO·Al_2_O_3_·Fe_2_O_3_. Types of cement defined in the Korean Industrial Standards (KS) are presented in Table 4 [10]. The properties of cement, such as hydration heat generation, hardening rates, and acid resistance to sulfates, vary depending on the type of cement.

The amount of water required to prepare cement mortar is determined according to the “Testing Method for Standard Consistency of Hydraulic Cement” (KS-L-5102) [11]. In this method, the standard consistency of cement paste is defined as when the needle of a Vicat tester moves from the surface of cement mortar down to a point 10 ± 1 mm below the surface within a period of 30 s. For Portland Cement Type I, the amount of water used in the cement hydration reaction can be stoichiometrically calculated using theoretical chemical reaction formulas. In reality, however, the actual amount of water chemically combined with cement is affected by the composition, hardening time, and *w*/*c* ratio of cement. Thus, a number of empirical formulas were developed based on actual experimental data. Those reported in previous studies are summarized in Table 5. The minimum amount of water per 100 g of cement required to prepare cement mortar varied depending on the estimation method: 29.64 g when stoichiometrically calculated, 24.81 g when calculated based on empirical Equations proposed by Kantro, and 24.0–27.0 g when calculated according to the standard consistency test method [12]. The minimum *w*/*c* ratio required for hydration reactions was found to be 0.25–0.30 with no consideration of the fluidity of the resultant mortar.

The minimum amount of water to prepare cement mortar per 100 g of cement was 24.0–27.0 g. Simply put, the minimum *w*/*c* ratio required for cement paste preparation was 0.24–0.27 with no consideration of the fluidity of the resultant paste.

#### 2.1.2. Waste Ion Exchange Resin (Spent Resin)

The specifications of Amberlite IRN-150 LC, a resin used in the present study, are summarized in Table 6 [13]. This type of resin has long been widely used in the nuclear power field. This mixed resin is prepared by mixing IRN-77, a cation exchange resin, and IRN-78, an anion exchange resin, at a volumetric ratio of 4:6. This mixing ratio is related to the exchange capacity of the respective resins used. Given that the anion exchange resin IRN-78 has a lower exchange capacity than the cation exchange resin IRN-77, the mixing ratio of IRN-78 is higher than that of IRN-77 so that the equivalent exchange capacity can be achieved for each segment. As shown in the table, the anion resin is slightly smaller in diameter than in the cation resin, and the density of the anion resin is also slightly lower compared to the cation resin. These features may cause micro delamination in the ion exchange process, for example, in resin towers or spent resin storage tanks, or during the cement solidification process of waste resins.

### 2.2. Experimental Methods and Evaluation

#### 2.2.1. Fabrication of Waste-Form Drums

The optimum operating conditions of the cement solidification process were determined considering various factors, including the integrity of waste forms, waste resin content for maximized cost-effectiveness, and operating errors of the solidification process, as follows: *w*/*c* ratio of 0.35 and waist resin content of 11 wt.%. These conditions fulfilled the acceptance criteria for low- and intermediate-level radioactive waste. Afterward, waste-form drums were fabricated in the cement solidification process including KORAD’s in-drum mixer using the optimum operating conditions for 30 min. The components and their respective ratios are presented in Table 7.

**Figure 2:**
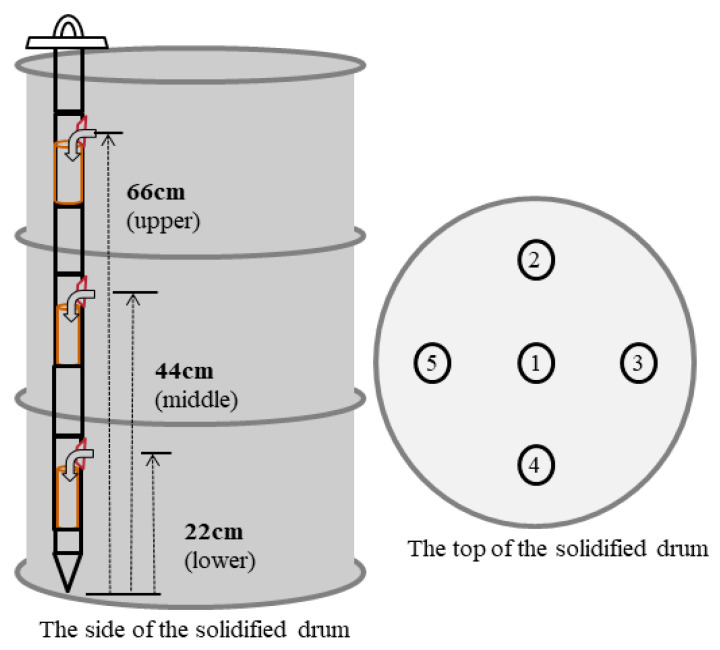
Five collection points on the top surface of the drum and another three points along the vertical direction (upper/middle/lower) for each collection point.

In the present study, representative samples were collected from waste-form drums fabricated in the cement solidification process within the set time frame, i.e., 3.5 h of the initial setting time of the drums, using a specially designed sample collector. This sample collector was designed to collect multiple samples at the same time along the vertical direction of each drum, i.e., from the upper (66 cm), middle (44 cm), and lower (22 cm) parts of each drum. The collector can be equipped with a polyethylene mold (φ of 5 cm and a height of 12 cm) at each of the three positions. Figure 2 shows five collection points for representative samples and another three points along the vertical direction of the drum (upper, middle, and lower positions) for each collection point. First, five points on the top surface of the drum were selected, including the center of the drum, and sample collectors were then inserted at each of the five points to collect three representative samples at the same time along the vertical direction of the drum, i.e., from the upper, middle, and lower parts, to ensure that the collected samples can properly represent the properties of the tested drum. As a result, a total of 15 samples were collected from each drum, i.e., three samples from each of the five points.

The disposal safety of waste forms was assessed by fabricating waste-form drums in the cement solidification process using the optimal operating conditions and then collecting representative samples from the obtained drums. Representative samples were collected as follows. Five collection points on the top surface of the drum were selected, including the center of the drum, and sample collectors were then inserted at each of the five points to collect three representative samples at the same time along the vertical direction of the drum, i.e., from the upper, middle, and lower parts. A set of three specimens collected from the upper, middle, and lower parts of each drum were subjected as a group to disposal safety assessments. The sample collection points, vertical-direction points for each collection point, and sample codes, along with test items, are summarized in Table 8. For leaching tests, three specimens were prepared at the laboratory level to ensure the accuracy of test results, and also because the required reagents were costly.

**Figure 3:**
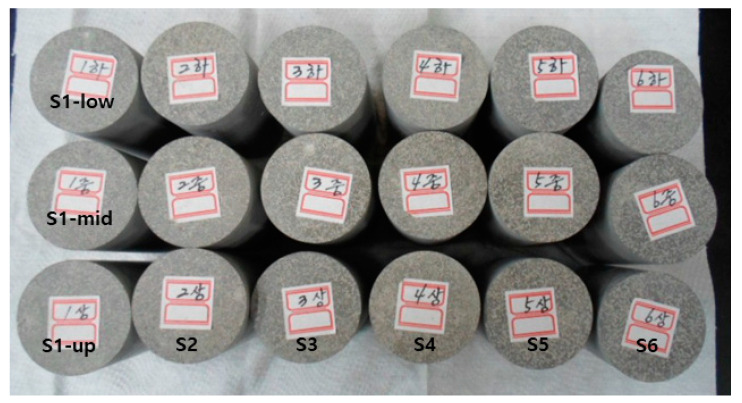
Image of polished representative samples collected from waste-form drums.

After the curing process, sealing lids were removed, and the samples were then tested for the presence of remaining free-standing water and surface cracks through visual inspections. Each sample was de-molded and then polished using fine sandpaper to make its top and bottom surfaces parallel. The measurement of compressive strengths, as well as the estimation of surface area and volume of each sample, may be affected by how parallel their top and bottom surfaces are. All this, in turn, affects the estimation of their leachability indexes for each nuclide. The physical properties of these processed samples, such as height, diameter, and weight, were then measured. Figure 3 shows an image of polished representative samples.

#### 2.2.2. Test Methods and Evaluation Criteria for Waste-Form Drums

Homogeneity is one of the most important factors that determine the quality of waste forms as a measure to evaluate whether the corresponding cement solidification process is properly performed. The homogeneity of a waste-form drum is evaluated by measuring the density of representative samples collected from the upper, middle, and lower parts of the drum. The samples were also evaluated for six test items provided in the WAC for low- and intermediate-level radioactive waste. All tests and evaluations were performed in accordance with the test methods and evaluation criteria described in the WAC.

The structural stability of waste forms is generally evaluated using early compressive strength tests, water immersion tests, thermal cycling tests, and irradiation tests. Each test was conducted on a set of three samples, and if any cracks or collapses were found in any of the three samples, the corresponding waste form was classified as nonconforming. Among the samples, those that were classified as conforming were then subjected to compressive strength tests. Those with 35.2 kgf/cm^2^ or more were evaluated as conforming. The leaching safety of waste forms was evaluated through leaching tests for a period of 90 days in accordance with ANS 16.1. Here, waste forms are required to have a leachability index of 6 or higher for each nuclide, i.e., Cs, Sr, and Co. The presence of free-standing water in waste forms was evaluated by conducting a free-standing water test on crushed samples. If the volume content of free-standing water was 0.5 vol.% or less, then the corresponding sample was classified as conforming. The acceptance criteria for low- and intermediate-level radioactive waste applied to the waste resin cement forms in the present study are presented in Table 9 [14].

#### 2.2.3. Preparation of Leaching Test Specimens and Leachability Index

Some researchers fabricated a contaminated resin by pouring an aqueous solution of Cs, Sr, and Co ions into a mixed resin, stirring the solution for 18 h, dehydrating the aqueous solution to obtain a dried resin, and finally cleaning the resin with demineralized water for five times. The researchers reported that the experimental sorption capacity of cation exchange resins for non-radioactive nuclides, such as Cs, Sr, and Co, was about 25% of the theoretical sorption capacity [16].

Leachability was examined via inactive tests. CsCl, SrCl_2_·6H_2_O, and CoCl_2_·6H_2_O were used as chemical compounds corresponding to Cs, Sr, and Co, respectively. The required amount of the respective compounds corresponding to each nuclide was determined stoichiometrically, and, accordingly, 2 L of an aqueous solution containing these compounds was prepared. This aqueous solution was poured into a prepared damp resin and then stirred at a low rate (54 rpm) for 24 h. Afterward, the contaminated resin was cleaned with demineralized water three times and then subjected to decompression dehydration. The obtained resin in a damp state was used in leaching tests. The amount of each nuclide absorbed in the contaminated resin (*A_o_*) was calculated using the following Equation, i.e., by subtracting the amount of the nuclide that remained in the aqueous solution from the total amount of the nuclide added in the aqueous solution.

(1) $$A_{o}=N_{i}-N_{e}$$

where *N_i_* is the total amount of the nuclide added to the aqueous solution, and *N_e_* is the amount of the nuclide that remained in the aqueous solution.

Three specimens were prepared for leaching tests, and the three measurements were averaged for analysis.

The cumulative fraction leached (CFL) can be expressed as Equation (2) below.

(2) $$\mathrm{CFL}=f\left(t\right)=\frac{\sum a_{n}}{A_{0}}=2\frac{S}{V}[\frac{D_{e}}{\pi }{]}^{1/2}\cdot t^{\frac{1}{2}}$$

where CFL = cumulative fraction leached, *a_n_* = total amount of the material released during leaching periods up to time *t*, *V* = waste form volume, *S* = surface area of the waste form, and *D_e_* = effective diffusion coefficient [cm^2^/s].

If $\left[\frac{\sum a_{n}}{A_{0}}\right]$ in Equation (2) is plotted against $\left[t^{\frac{1}{2}}\right]$,

(3) $$\mathrm{slope}=2\frac{S}{V}[\frac{D_{e}}{\pi }{]}^{\frac{1}{2}}$$

The slope can be determined from the graph, and based on the result, the *D_e_* can be estimated using Equation (4) below.

(4) $$D_{e}={\left(\mathrm{slope}\right)}^{2}\cdot [\frac{1}{2}\frac{V}{S}{]}^{2}\cdot \pi$$

The leaching safety of the representative samples of waste forms was evaluated in terms of the leachability index (LX) specified in the WAC. The LX is defined as Equation (5).

(5) $$\mathrm{LX}=-\log D_{e}$$

## 3. Results and Discussion

### 3.1. Evaluation of Homogeneity and Structural Safety of Waste-Form Drums

#### 3.1.1. Evaluation of Homogeneity of Waste-Form Drums

Homogeneity is an important factor that determines the quality of waste forms as a measure to evaluate whether the corresponding cement solidification process is properly performed, especially as to the performance of stirrers and the appropriateness of stirring time. In the present study, homogeneity was evaluated by measuring and comparing the density of representative samples collected from the upper, middle, and lower parts of each waste-form drum. A total of 15 representative samples collected from the upper (66 cm), middle (44 cm), and lower (22 cm) parts of each drum were tested. The average densities measured at the upper, middle, and lower parts of the waste-form drums were at a similar level, that is, 1.853, 1.853, and 1.849 g/cm^3^, respectively, and thus these waste-form drums were considered homogeneous.

#### 3.1.2. Structural Safety of Waste Forms

**Figure 4:**
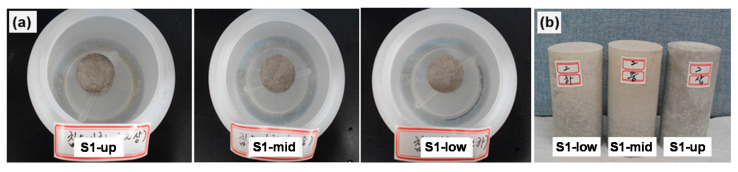
(**a**) Images of representative samples subjected to the water immersion test and (**b**) Samples after the test.

The structural safety of the waste forms was evaluated by measuring their compressive strength after the early compressive strength tests, thermal cycling tests, water immersion tests, and irradiation tests. The obtained samples were crushed before being subjected to free-standing water tests. Figure 4 shows images of representative samples of the waste-form drums subjected to the water immersion test (a) and the samples after the test (b).

**Figure 5:**
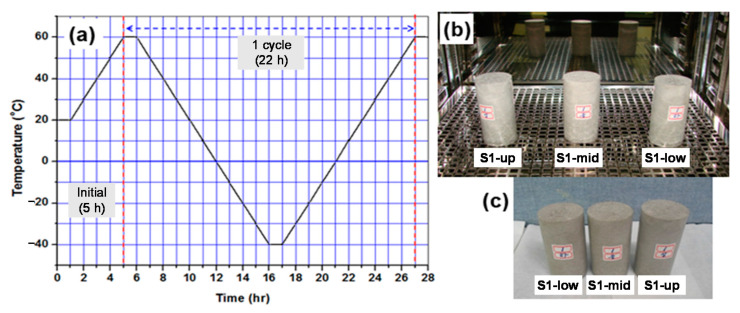
(**a**) Illustration of temperature changes during a single cycle (**b**) Image of the samples subjected to the test (**c**) Image of the samples after the test.

A single cycle in thermal cycling testing consists of a cooling step from 60 °C to −40 °C, followed by a heating step from −40 °C back to 60 °C at a rate of 10 °C/h over a total period of 22 h. These thermal cycling tests were repeated for a total of 30 cycles, and it took about 28 days. Following the thermal cycling tests, the waste form samples were then subjected to a visual inspection for any cracks or other defects, and then their compressive strength was measured. Figure 5 shows an illustration of temperature changes during a single cycle (a) and images of the samples subjected to the test (b) and after the test (c).

**Figure 6:**
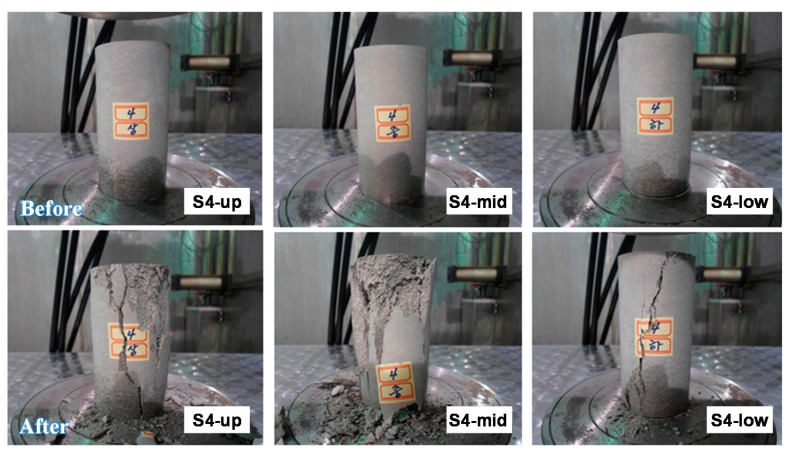
Irradiated samples before and after the compressive strength tests.

It was reported that waste forms disposed of at low- and intermediate-level radioactive waste disposal sites are exposed to up to 1.0 × 10^6^ Gy (1.0 × 10^8^ rad) of radiation from sources, such as radionuclides contained in radioactive waste over the course of 300 years of the required management period. Irradiation tests were performed at atmospheric pressure (1 atm) and room temperature (17.8 °C), and Co-60 (210,536 Ci) was used as a radiation source to produce gamma rays. The total absorbed dose for each test sample was set to 1.0 × 10^6^ Gy (1.0 × 10^8^ rad). Figure 6 shows images of the irradiated samples before and after the compressive strength tests.

**Figure 7:**
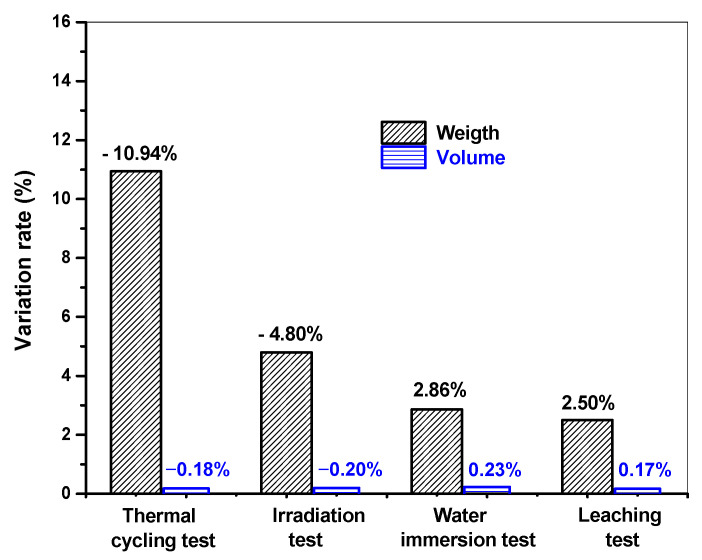
Changes in the weight and volume of the representative samples before and after each test.

**Figure 8:**
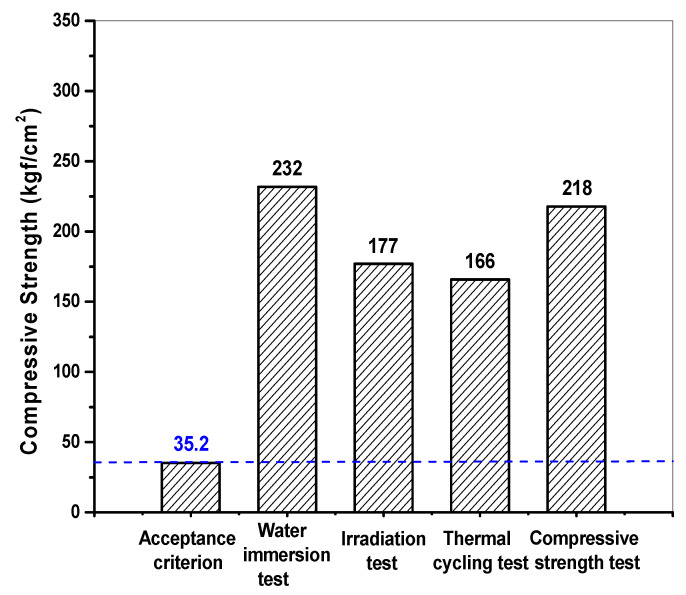
Comparison of compressive strengths of the representative samples of waste-form drums after each test (0.35 of *w*/*c* and 11 wt.% of spent resin).

Figure 7 presents changes in the weight and volume of waste form samples after the thermal cycling tests, irradiation tests, water immersion tests, and leaching tests. After each of these tests, the samples were stored in a curing room for two days, and then their weight and volume were measured. The weight of the waste form samples decreased by 10.94% and 4.80% after the thermal cycling tests and irradiation tests, respectively. In contrast, the weight increased by 2.86% and 2.50% after the water immersion tests and leaching tests, respectively. The observed changes in the weight of the samples were attributed to dehydration or water absorption selectively occurring in the samples depending on the nature of each test. It was also found that, even after the samples were stored in a curing room for two days, their weight did not return to their initial levels. The respective volume changes observed in the samples after each test were all 0.23% or less and thus were considered measurement errors. The measured properties of the cement waste form samples were assessed against the WAC, as shown in Figure 8. The compressive strengths of each sample ranged from 166 to 232 kgf/cm^2^, which are greater than the threshold provided in the WAC at 35.2 kgf/cm^2^ (3.44 MPa). This result confirmed that the waste-form drums provided sufficient structural stability for disposal considerations.

### 3.2. Leaching Safety of Waste Forms

**Figure 9:**
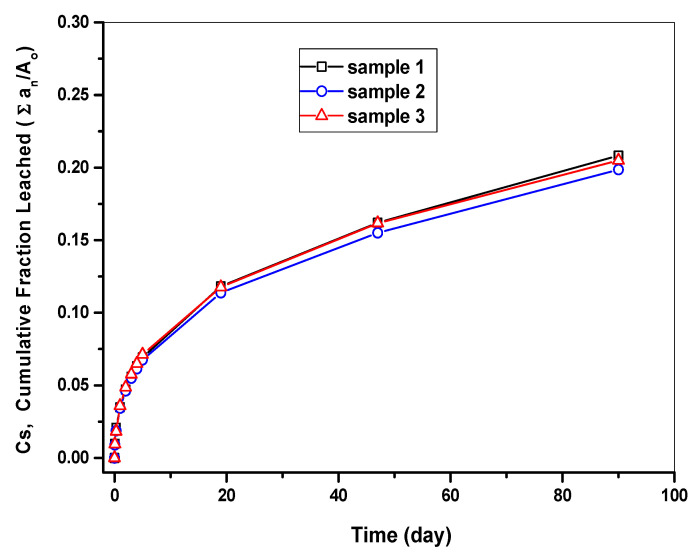
Cumulative fraction leached of Cs measured from the cement waste form.

The respective CFLs of each nuclide (Cs, Sr, and Co) are presented in Table 10. Figure 9 presents changes in the CFL of Cs over time (day) measured in the cement waste form samples. It was found that the leaching of Cs occurred at a rapid pace for the first five days of the test and then started to slow down over time; the CFL became saturated over time. All three specimens were found to have a very similar CFL.

**Figure 10:**
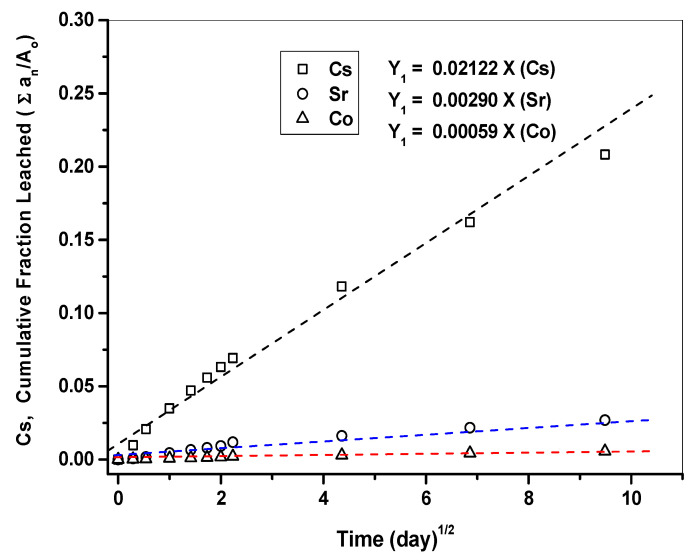
Respective CFLs for each nuclide (Cs, Sr, and Co), along with the slopes of each curve.

**Figure 11:**
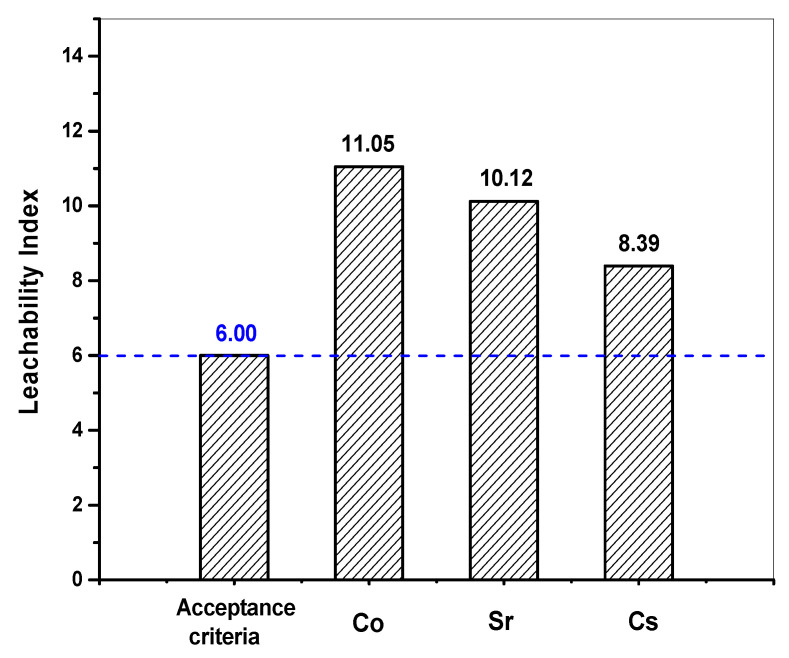
Leaching indexes for each nuclide compared against the WAC.

Figure 10 presents the respective CFLs of each nuclide (Cs, Sr, Co) as a function of time measured from the cement waste form samples, along with the slopes of each curve. The higher the slope is, the greater the degree of leaching becomes. Accordingly, the degree of leaching was found to decrease in the order of Cs > Sr > Co. These results agree with the *D_e_* and CFL values for each nuclide during the test. In addition, the more linear the curve is, the more consistent the leaching of nuclides is with the typical diffusion model. Table 11 shows the respective LX calculated based on the *D_e_* for each nuclide (Cs, Sr, and Co) using Equation (5). The LX values calculated for each nuclide (Cs, Sr, and Co) were then evaluated against the WAC, as shown in Figure 11. The LX values for each nuclide were found to be all greater than six, the threshold provided in the WAC. These results confirmed that the waste-form drums provided sufficient leaching safety for disposal.

### 3.3. Free-Standing Water Tests of Waste-Form Drums

**Figure 12:**
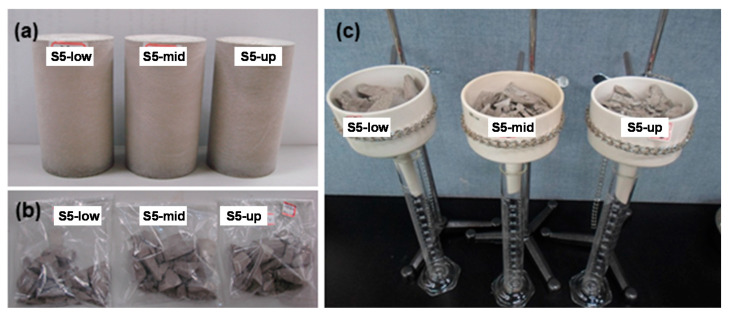
(**a**) Free-standing water test specimens, (**b**) Crushed specimens, and (**c**) Experimental settings for the test.

The volume content of free-standing water in waste forms must be 0.5 vol.% or less. In the present study, three representative specimens collected from the waste-form drums were used in free-standing water tests. The diameter and height of the three specimens were measured using vernier calipers to estimate their volume. Before being tested, the specimens were crushed to ensure that free-standing water contained inside them could be easily released. Figure 12 shows images of free-standing water test specimens (a), crushed specimens (b), and the experimental settings for the test (c). The results confirmed that free-standing water had not been generated (all specimens show 0.0 vol.%). Overall, in the present study, the waste-form drums were tested for the six test items provided in the WAC for low- and intermediate-level radioactive waste, and the results confirmed that the drums adequately met the requirements of the six test items provided in the WAC.

## 4. Conclusions

The present study aimed to evaluate the utility of the cement solidification process for stably disposing of waste ion exchange resin generated during the treatment of radioactive wastewater. The optimum operating conditions were determined in consideration of both cost-effectiveness and safety: the optimum *w*/*c* ratio was set to 0.35, and the optimum resin content was determined to be 11 wt.%. Waste-form drums were produced in the cement solidification process using the determined optimum operation conditions, and representative samples were collected from the drums and used to assess their disposal safety. The disposal safety of waste forms was evaluated according to the waste acceptance criteria (WAC) applicable to domestic waste disposal sites, and the tests were conducted for six test items provided in the WAC. A total of 15 representative samples were collected from the waste-form drums produced using the optimum operating conditions, and their structural stability was evaluated to satisfy disposal conditions. In addition, the LX of all samples tested in the present study exceeded the WAC (>6) for the disposal of radioactive wastes. The results confirmed that these waste-form drums adequately met the requirements of each test item provided in the WAC. Therefore, cement waste forms including waste ion exchange resin produced through this process were considered to be conforming to the requirements for disposal safety.

## Figures and Tables

**Table 1:** Classification of ion-exchange resins according to the moisture content.

Items	Properties
As-received resins	Ion exchange resins in an as-received state Water uptake of resins: moisture content inside the resin ≤ Saturation level
Damp resins	Only the inside part is saturated with moisture Prepared by immersing resins in distilled water for 18 h or more and then filtering them in a decompressed state at 5–10 psi for at least 10 min With electrostatic free-standing water present near resin particles removed
Dewatered resins	With free-standing water drained by decanting or using drain valves With moisture and electrostatic free-standing water inside the resin remaining
Slurry resins	Resins in a state that can be delivered using a pump-Delivery: Possible when the content of free-standing water is 30% or more

**Table 2:** Requirements to consider when selecting a solidification process.

Requirements	Selected Processes
(1) Simplicity of the process (2) Reliability of the process (3) Easy maintenance (4) Minimum radiation exposure to workers (5) Reasonable cost	Cementation process— In-drum system
(6) Systematic procedures - Quality assurance of waste form - Consistency of quality	PCP (a systematic process control program)

**Table 3:** Factors to consider when operating a solidification process facility.

Items	Content	Comments
Stability of waste forms	Compressive strength	For cement waste forms -Critical factor: fragmentation during water immersion tests
	Immersion	
	Thermal circulation	
	Irradiation	
	Free-standing water	
	Leaching resistance	
Economic aspects		Maximum loading of waste
Process characteristics	Quantification of mixing ratios	Durability of a stirrer Water uptake of waste resin
	Workability of mixtures	
	Homogeneity of waste forms	

**Table 4:** Composition (wt%) of Portland cement (KS) [10].

Type	C_3_S ^(1)^	C_2_S ^(2)^	C_3_A ^(3)^	C_4_AF ^(4)^	Others	Characteristics
I	45	27	11	8	9	Normal
II	44	31	7	13	5	Modified
III	53	19	10	7	11	High early strength
IV	20	52	6	14	8	Low heat
V	38	43	4	8	7	Sulfate resistant

^(1)^ 3CaO·SiO_2_, ^(2)^ 2CaO·SiO_2_, ^(3)^ 3CaO·Al_2_O_3_, and ^(4)^ 4CaO·Al_2_O_3_·Fe_2_O_3_.

**Table 5:** Minimum *w*/*c* ratio required for hydration reactions of Portland Cement Type I.

Item	Required Amount of Water (Per 100 g of Cement)	*w*/*c* Ratio
Theoretical and stoichiometric estimation	29.64 g	About 0.30
Empirical Equations by Kantro [12]	24.81 g	About 0.25
Standard consistency test *	24.0–27.0 g **	About 0.25

* Korean Industrial Standards (KS); ** Commercial cement products from various domestic cement manufacturers.

**Table 6:** Specifications of Amberlite IRN-150LC resin used in the tests [13].

Properties	IRN-150 LC	
	IRN-77 (Cation)	IRN-78 (Anion)
Parent resin	IRN-120	IRA-400
Ionic form	H^+^	OH^−^
Particle size (mm)	0.3–1.2	0.3–1.2
(Mean size)	(0.6–0.7)	(0.58–0.68)
Moisture content (wt.%)	49–55	55–60
Exchange capacity (meq/mL)	1.9	1.2
Mixed vol. ratio	4	6
pH	10.3	8.5

IRN-150: Rohm and Haas Company.

**Table 7:** Components of cement waste forms fabricated using the optimal operating conditions and their respective ratios.

Cement	Ratio	Ratio (wt.%)
Portland Cement Type I	Water/cement	Spent resin/cement
	0.35	11

**Table 8:** Sample codes, sample collection points, and vertical-direction positions, along with test items.

Items	Sample Name	Points along the Vertical Direction	Collection Points
Thermal cycling test	S1-upper	Upper (66 cm)	1
	S1-middle	Middle (44 cm)	
	S1-lower	Lower (22 cm)	
Water immersion test	S2-upper	Upper (66 cm)	2
	S2-middle	Middle (44 cm)	
	S2-lower	Lower (22 cm)	
Compressive strength test	S3-upper	Upper (66 cm)	3
	S3-middle	Middle (44 cm)	
	S3-lower	Lower (22 cm)	
Irradiation test	S4-upper	Upper (66 cm)	4
	S4-middle	Middle (44 cm)	
	S4-lower	Lower (22 cm)	
Free-standing water test	S5-upper	Upper (66 cm)	5
	S5-middle	Middle (44 cm)	
	S5-lower	Lower (22 cm)	
Leaching test	S6-1	Laboratory manufacturing	
	S6-2		
	S6-3		

**Table 9:** Test items and methods specified in KORAD’s acquisition criteria [14].

Item	Test	Standard Method	Test Method	Criteria
Structural stability	Compressive strength test	KS F2405	-	≥35.2 kgf/cm^2^ (3.44 MPa)
	Water immersion test (90 days)	NRC *	Compressive strength after immersion tests	≥35.2 kgf/cm^2^
	Thermal cycling test (28 days)	ASTM B553	Compressive strength after thermal cycling tests	≥35.2 kgf/cm^2^
	Irradiation test	NRC *	Compressive strength after irradiation tests (1.0 × 10^6^ Gy)	≥35.2 kgf/cm^2^
Leachability	Leaching test (90 days)	ANS 16.1	Cs, Sr, Co	Leachability Index ≥ 6
Free standing water test	Sample	EPA **	-	<0.5 vol.%
	200 L/drum	ANS 55.1	-	<0.5 vol.%

* NRC, Waste Form Technical Position, Revision 1. (1991); ** EPA, Method 9095B “Paint Filter Liquids Test” [15].

**Table 10:** Cumulative fraction leached of each nuclide (Cs, Sr, and Co) measured from the cement waste form.

No.	Σ(Δ*t*)_n_ (Day)	Σ(Δ*t*)_n_ (Day)^1/2^	Cumulative Fraction Leached [∑*a_n_*/*A_o_*]		
			Cs	Sr	Co
1	0.083	0.288	9.43 × 10^−3^	6.16 × 10^−4^	2.20 ×10^−4^
2	0.292	0.540	1.93 × 10^−2^	1.70 × 10^−3^	4.41 × 10^−4^
3	1	1.000	3.50 × 10^−2^	3.97 × 10^−3^	7.35 × 10^−4^
4	2	1.414	4.73 × 10^−2^	6.09 × 10^−3^	1.03 × 10^−3^
5	3	1.732	5.62 × 10^−2^	7.55 × 10^−3^	1.36 × 10^−3^
6	4	2.000	6.32 × 10^−2^	8.83 × 10^−3^	1.69 × 10^−3^
7	5	2.236	6.94 × 10^−2^	1.13 × 10^−2^	2.09 × 10^−3^
8	19	4.359	1.17 × 10^−1^	1.55 × 10^−2^	2.98 × 10^−3^
9	47	6.856	1.60 × 10^−1^	2.16 × 10^−2^	4.30 × 10^−3^
10	90	9.487	2.04 × 10^−1^	2.75 × 10^−2^	5.62 × 10^−3^

**Table 11:** Effective diffusion coefficient and leaching indexes for each nuclide (Cs, Sr, and Co).

Nuclide	Sample No.	V/S (cm)	Slope	*D_e_* (cm^2^/day)	*D_e_* (cm^2^/s)	Leachability index
Co	S 1	0.999	6.0773 × 10^−4^	2.8973 × 10^−7^	3.3534 × 10^−12^	11.47
	S 2	1.000	5.9188 × 10^−4^	2.7481 × 10^−7^	3.1807 × 10^−12^	11.50
	S 3	0.999	5.6743 × 10^−4^	2.5257 × 10^−7^	2.9233 × 10^−12^	11.53
	Average	0.999	5.8901 × 10^−4^	2.7237 × 10^−7^	3.1524 × 10^−12^	11.50
Sr	S 1	0.999	2.8500 × 10^−3^	6.3717 × 10^−6^	7.3747 × 10^−11^	10.13
	S 2	1.000	2.8400 × 10^−3^	6.3296 × 10^−6^	7.3260 × 10^−11^	10.14
	S 3	0.999	3.0100 × 10^−3^	7.1072 × 10^−6^	8.2260 × 10^−11^	10.08
	Average	0.999	2.9000 × 10^−3^	6.6029 × 10^−6^	7.6422 × 10^−11^	10.12
Cs	S 1	0.999	2.1650 × 10^−2^	3.6769 × 10^−4^	4.2557 × 10^−9^	8.37
	S 2	1.000	2.0650 × 10^−2^	3.3451 ×10^−4^	3.8716 × 10^−9^	8.41
	S 3	0.999	2.1350 × 10^−2^	3.5757× 10^−4^	4.1386 × 10^−9^	8.38
	Average	0.999	2.1217 × 10^−2^	3.5326 × 10^−4^	4.0886 × 10^−9^	8.39

## Data Availability

The data presented in this study are available on request from the corresponding author. The data are not publicly available due to institutional and national data-sharing restrictions.
